# 

**Published:** 1902-06-15

**Authors:** 


					﻿THE
DENTAL REGISTER
EDITED BY
NELVILLE S. HOFF, D. D. S.,
ANN ARBOR, MICH.
Vol. LVI.	JUNE 15, 1902.	No. 6.
PRICE, ONE DOLLAR A YEAR im.advxmc^
PUBLISHED MONTHLY BY
SAMUEL A. CROCKER & CO.,
THE OHIO DENTAL AND SURGICAL DEPOT.
¡5“ to 31) 'W. “tli St., C iueimiiiti. O.
Entered at the Postofiice at Cincinnati, 0., as second-class mail matter.
				

